# Posttraumatic Stress Disorder Is Associated with α Dysrhythmia across the Visual Cortex and the Default Mode Network

**DOI:** 10.1523/ENEURO.0053-20.2020

**Published:** 2020-07-31

**Authors:** Kevin J. Clancy, Jeremy A. Andrzejewski, Jessica Simon, Mingzhou Ding, Norman B. Schmidt, Wen Li

**Affiliations:** 1 Department of Psychology, Florida State University, Tallahassee, FL 32304; 2 J. Crayton Pruitt Family Department of Biomedical Engineering, University of Florida, Gainesville, FL 32611

**Keywords:** α oscillations, default mode network, posttraumatic stress disorder, resting state, sensory disinhibition

## Abstract

Anomalies in default mode network (DMN) activity and α (8–12 Hz) oscillations have been independently observed in posttraumatic stress disorder (PTSD). Recent spatiotemporal analyses suggest that α oscillations support DMN functioning via interregional synchronization and sensory cortical inhibition. Therefore, we examined a unifying pathology of α deficits in the visual-cortex-DMN system in PTSD. Human patients with PTSD (*N *=* *25) and two control groups, patients with generalized anxiety disorder (GAD; *N *=* *24) and healthy controls (HCs; *N *=* *20), underwent a standard eyes-open resting state (S-RS) and a modified resting state (M-RS) of passively viewing salient images (known to deactivate the DMN). High-density electroencephalogram (hdEEG) were recorded, from which intracortical α activity (power and connectivity/Granger causality) was extracted using the exact low-resolution electromagnetic tomography (eLORETA). Patients with PTSD (vs GAD/HC) demonstrated attenuated α power in the visual cortex (VC) and key hubs of the DMN [posterior cingulate cortex (PCC) and medial prefrontal cortex (mPFC)] at both states, the severity of which further correlated with hypervigilance symptoms. With increased visual input (at M-RS vs S-RS), patients with PTSD further demonstrated reduced α-frequency directed connectivity within the DMN (PCC→mPFC) and, importantly, from the VC to both DMN hubs (VC→PCC and VC→mPFC), linking α deficits in the two systems. These interrelated α deficits align with DMN hypoactivity/hypoconnectivity, sensory disinhibition, and hypervigilance in PTSD, representing a unifying neural underpinning of these anomalies. The identification of visual-cortex-DMN α dysrhythmia in PTSD further presents a novel therapeutic target, promoting network-based intervention of neural oscillations.

## Significance Statement

α (8–12 Hz) oscillations and the default mode network (DMN) both dominate the resting-state brain activity and are found to be closely related. In addition, aberrant α and DMN activities are both implicated in the pathophysiology of posttraumatic stress disorder (PTSD). Linking α and DMN aberrations in PTSD, our high-density electroencephalogram (hdEEG) source analysis reveals that PTSD is associated with α power deficits across the DMN and visual cortex (VC) and deficient α-frequency connectivity from the VC to the DMN. That this visual-cortex-DMN α dysrhythmia further underpins hypervigilance symptoms in PTSD highlights a temporal-spatial network pathology, promoting network-based neural oscillatory interventions.

## Introduction

Prevailing models of the neuropathology of posttraumatic stress disorder (PTSD) have focused on dysfunctions of the prefrontal-cortex-amygdala circuit (Rauch et al., 2006; Liberzon and Abelson, 2016). Recently, evidence has extended this circuit pathology to implicate large-scale brain network anomalies (Spielberg et al., 2015; Koch et al., 2016; Liberzon and Abelson, 2016; Akiki et al., 2017). Anomalies in the default mode network (DMN), a major resting-state network (RSN), have been especially highlighted in this literature, characterized by attenuated network activity and disrupted network communication (Bluhm et al., 2009; Lanius et al., 2010; Sripada et al., 2012; Koch et al., 2016; Akiki et al., 2018).

The DMN is one of the most consistently identified RSNs, anchored in a midline core consisting of two key hub structures, the posterior cingulate cortex (PCC) and the medial prefrontal cortex (mPFC; Buckner et al., 2008; Yeo et al., 2011). While prominent in the resting state, the DMN is deactivated by salient sensory input or externally-oriented cognitive processing and, accordingly, exhibits reciprocal inhibition with neural networks associated with these processes (Gusnard et al., 2001; Raichle et al., 2001; Greicius et al., 2003; Greicius and Menon, 2004). As such, DMN dysfunctions can interrupt internal mentation or ‘tranquil’ resting states and heighten vigilance and attention. Indeed, DMN dysfunction has been linked to heightened acute stress response (Menon, 2011; Hermans et al., 2014; Zhang et al., 2019) and implicated in PTSD symptoms of hypervigilance and negative intrusions (Abdallah et al., 2017; Akiki et al., 2018).

While neuroimaging data have isolated the DMN as the dominant network in the resting brain, electrophysiological data have identified α (8–12 Hz) oscillations as the dominant electrical activity in the resting brain (Klimesch et al., 2007; Klimesch, 2012). α Oscillations represent a neural mechanism mediating long-range interregional interactions (Palva and Palva, 2007; Tang et al., 2007; Hillebrand et al., 2016). Importantly, evidence has begun to link α oscillations to DMN activity (especially during eyes-open resting state; Mantini et al., 2007; Jann et al., 2009; Knyazev et al., 2011; Scheeringa et al., 2012; Mo et al., 2013), raising the possibility that, to some extent, the DMN could be organized and maintained by long-range synchronization of α oscillations (Engel and Singer, 2001; Uhlhaas et al., 2008; Jann et al., 2009).

While α oscillations positively correlate with DMN activity, they are known to negatively correlate with visual cortical activity (Klimesch et al., 2007; Jensen and Mazaheri, 2010; Lange et al., 2013). α Oscillations (originating in the sensory cortex and thalamus) play a key role in visual inhibition by suppressing cortical excitation and feedforward propagations (Klimesch et al., 2007; Palva and Palva, 2007; Tang et al., 2007; Jensen and Mazaheri, 2010; Foxe and Snyder, 2011; Klimesch, 2012; Hillebrand et al., 2016; Johnson et al., 2017). This active function of α oscillations can present a second mechanism, visual cortical inhibition, to support DMN activity. That is, by suppressing visual processing, α oscillations could protect the DMN from environmental disruptions.

Alternatively, deficient α activity could give rise to DMN dysfunctions by failing to sustain long-range synchronization across the DMN and failing to inhibit sensory afferents to the DMN. Aberrant α oscillations have been featured in a “thalamocortical dysrhythmia” model of neuropsychiatric disorders (Llinás et al., 1999; Schulman et al., 2011), which are conceptualized transdiagnostically as oscillopathies (Basar, 2013; Buzsáki et al., 2013). Reduced resting-state α activity has been observed in patients with PTSD [vs healthy controls (HCs) and patients with generalized anxiety disorder (GAD); Clancy et al., 2017] and combat veterans with severe symptoms (Clancy et al., 2020). Given the demonstrated association between α oscillations and DMN activity, we hypothesized that PTSD is associated with deficient α activity in the DMN and deficient inhibition of sensory cortical input to the DMN.

Therefore, by extracting intracortical α activity from high-density electroencephalogram (hdEEG) recordings, we conducted source-level analysis of resting-state α activity in patients with PTSD, relative to HCs and patients with GAD. The GAD group was included to rule out effects of general anxiety and hyperarousal that would confound resting-state neural oscillations (Imperatori et al., 2019). For simplicity and statistical rigor, the GAD group and HC group were collapsed into a single control group, which was further justified by prior work by (Clancy et al. (2017), demonstrating a lack of surface-level differences between HC and GAD groups. Comparisons between PTSD and HC or GAD groups separately are also reported. To highlight the vulnerability of the DMN to sensory input, we included a modified resting state (M-RS) involving passive viewing of images, in addition to a standard, eyes-open resting state (S-RS). We assessed two specific hypotheses of α deficits in PTSD, including (1) attenuated α power and connectivity in the DMN and (2) a deficit of α inhibition of visual cortical (VC) activity (i.e., attenuated α power) and attenuated directed α-frequency connectivity to the DMN (VC→DMN). Finally, we hypothesized that deficient sensory inhibition could be associated with the PTSD symptom of hypervigilance (characterized by excessive sensory scanning of the environment for threat) and thus examined these clinical associations, linking neuropathology to clinical symptomatology.

## Materials and Methods

### Participants

Participants consisted of outpatients with a current diagnosis of PTSD (*N *=* *25) or GAD (*N *=* *24), and HCs (*N *=* *20) with no current or past-year diagnoses. Participants were matched for age and gender across groups. Participants were recruited through community advertisement as part of a larger randomized controlled trial and compensated monetarily for their participation in the study. Inclusion criteria were primary diagnoses of PTSD or GAD, respectively, or no current or past-year psychiatric disorders for the HC group. Participants with co-morbid diagnoses of PTSD and GAD were excluded. Participants were further excluded based on the following criteria: history of suspected severe TB1 (i.e., exceeding 30 min of loss of consciousness because of a head or neck injury), history of neurologic disorders, and diagnosis of psychotic disorders, severe substance use disorder, or abuse of opioids, stimulants, or cocaine. Six PTSD participants met diagnostic criteria for mild alcohol use disorder (*n* = 3) or mild cannabis use disorder (*n* = 3); 32% of controls (GAD *n *=* *7, HC *n *=* *7) had a history of a DSM-5 criterion A trauma. Index trauma types of participants with PTSD ranged from combat exposure (*n *=* *6) and vehicular accident (*n *=* *3) to rape (*n *=* *7) and sexual (*n *=* *5) or physical assault (*n *=* *4); 48% (*n *=* *12) of participants in the PTSD group reported multiple traumas. The mean number of traumas in the PTSD group was 4.22 (±2.26). All participants provided written, informed consent to participate in the study, which was approved by both the university’s Institutional Review Board and the Department of Defense Human Research Protection Official’s Review. Demographic details are presented in Table 1.

**Table 1:** Participant demographics

	PTSD	Controls
Age (years)	34.6 ± 10.4	31.1 ± 13.1
Gender (female/male)	16/9	25/19
Substance use (%)^†^	64%^*^	7.5%
Medication use (%)	40%	36%
PCL total	61.0 ± 16.1^*^	37.6 ± 13.4
PCL-hypervigilance	4.1 ± 1.0^*^	2.1 ± 1.2
BAI	26.2 ± 15.7^*^	11.8 ± 9.4
BDI	26.8 ± 12.5^*^	17.4 ± 9.7

PCL = posttraumatic stress disorder checklist; BAI = Beck anxiety inventory; BDI = Beck depression inventory.

† = Subjects with opioid, stimulant, and cocaine use were excluded.

* *p *<* *0.005.

### Clinical assessment

Current or past-year diagnoses were assessed by trained clinicians using the Structured Clinical Interview for DSM-5 (American Psychiatric Association, 2013). Participants additionally completed the PTSD Checklist for DSM-IV: civilian version (Blanchard et al., 1996). In the present study, internal consistency was high for the total questionnaire (α = 0.95) and the hyperarousal subscale (α = 0.84). As in previous work (Mueller-Pfeiffer et al., 2013; Clancy et al., 2017), item 16 assessing symptoms of hypervigilance or “being watchful or on guard” was extracted to index hypervigilance.

### Experimental paradigm

Participants were seated in a comfortable recliner in a dimly lit, sound attenuated and electrically shielded room. hdEEG data were recorded during two resting states. To evaluate intrinsic neural activity, a standard resting state (S-RS) recording was conducted first, lasting 2 min while participants fixated on a crosshair on the screen. To assess the impact of environmental sensory input on α oscillations and DMN functioning, a M-RS recording was also conducted, involving 5 min of passively viewing a continuous stream of images (subtending a visual area of 7.8° × 5.8°), each for 333 ms. Images were chosen from the International Affective Picture System (Lang et al., 2008), depicting neutral (e.g., buildings, daily objects; *n* = 322), positive (e.g., erotic; *n* = 253), and negative (e.g., mutilation; *n* =346) scenes, randomly intermixed.

### EEG acquisition and preprocessing

EEG data were recorded from a 96 channel BrainProducts actiCap system with Neuroscan SynAmps RT amplifiers (1000-Hz sampling rate, 0.05- to 200-Hz online bandpass filter, referenced to the FCz channel). Electrooculogram (EOG) was recorded using four electrodes with vertical and horizontal bipolar derivations. EEG/EOG data were downsampled to 250 Hz, high-pass (1 Hz) and notch (60 Hz) filtered. We then applied *Fully Automated Statistical Thresholding for EEG artifact Rejection* algorithm (FASTER; Nolan et al., 2010) for artifact detection, correction, and rejection. Output data were epoched into 1-s segments and submitted to eLORETA for source analyses.

### Exact low-resolution electromagnetic tomography (eLORETA)

Using the high-density, artifact-minimized EEG data, we conducted intracranial source analyses using eLORETA, a linear inverse solution to reconstruct cortical activity with scalp EEG data (Pascual-Marqui et al., 2011). The LORETA algorithm has been cross-validated in multiple studies combining EEG-based LORETA with fMRI (Worrell et al., 2000; Vitacco et al., 2002; Mulert et al., 2004; Mobascher et al., 2009; Olbrich et al., 2009), positron emission tomography (Dierks et al., 2000; Pizzagalli et al., 2004), and intracranial recordings (Zumsteg et al., 2005). The solution space consists of 6239 cortical gray matter voxels with a spatial resolution of 5 × 5 × 5 mm in a realistic head model. eLORETA is a suitable tool to investigate network activity and connectivity (Neuner et al., 2014; Thatcher et al., 2014; Liu et al., 2017; Samogin et al., 2019) and has provided important network insights into psychiatric disorders (Whitton et al., 2018; Imperatori et al., 2019; Samogin et al., 2019).

For accurate inverse solutions, eLORETA was modeled for the S-RS and M-RS separately, from which whole-brain source estimates of α (8–12 Hz) power were derived (Pascual-Marqui et al., 2011). For source-based α-frequency connectivity analysis, time series of regions of interest (ROIs) were derived from eLORETA (Samogin et al., 2019), which were then submitted to Granger causality analysis based on bivariate autoregressive (AR) modeling (Ding et al., 2006). A model order of 20 (80 ms in time for a sampling rate of 250 Hz) was chosen in a two-step process: (1) Akaike Information Criterion (AIC) and (2) comparing spectral estimates obtained by the Fourier-based AR model for data pooled across all subjects (Wang et al., 2016). Our focus on the α frequency was guided by a priori hypotheses stemming from (1) the purported role of α oscillations in the sensory disinhibition model of PTSD and (2) prior work by Clancy et al. (2017), demonstrating no surface-level effects of PTSD-related aberrations in neighboring θ or β frequencies (*p*s > 0.16).

For ROIs, DMN hubs (i.e., PCC and mPFC) and visual cortex (VC) were selected as ROIs (Yeo et al., 2011; Tamber-Rosenau et al., 2013). ROIs were defined by gray-matter voxels within a 10-mm radius of the ROI centroids (Pascual-Marqui et al., 2011; Whitton et al., 2018). All ROIs were centered on the midline to incorporate both hemispheres, with centroid coordinates obtained from the Neurosynth (https:// www.neurosynth.org) meta-analysis maps (as peak voxels) of “default mode” (for the PCC: 0, −50, 30 and the mPFC: 0, 50, 0) and “passive viewing” (for the VC: 0, −90, 20). All coordinates are reported in Montreal Neurologic Institute (MNI) space. For connectivity analysis, both directions for each pair were examined such that the three ROIs resulted in a 3 × 3 matrix of α-GC connectivity for each of the two resting states.

### Statistical analyses

Source-level power and GC were submitted to planned simple contrasts of states (S-RS vs M-RS) to demonstrate the extent of α adaption from S-RS to M-RS. We then performed simple contrasts of group (PTSD vs control) for the two states to test PTSD-related deficits in α power and GC, and double contrasts of state and group to assess the effect of visual stimulation (S-RS minus M-RS) between groups. Pearson correlations were performed to assess clinical associations of α power and GC with symptom severity of hypervigilance. Guided by previous surface-level analyses showing no difference between the two control groups (GAD and HC; Clancy et al., 2017), we combined them into a single control group. Our supplemental analyses confirmed that these two control groups did not differ in source-level α activity.

Multiple comparison corrections were applied for the analyses. For power analyses involving whole-brain voxel-wise comparisons, we used Monte Carlo simulations (with actual Gaussian filter widths extracted from the data) to derive the corrected threshold (*p *<* *0.05): voxel level *p *<* *0.005 (one-tailed) over 11 contiguous voxels. As for connectivity analyses, the 3 × 3 connectivity matrix resulted in six comparisons for each hypothesis testing, for which we applied the false discovery rate (FDR) criterion (FDR *p *<* *0.05). Lastly, for clinical association analyses, we conducted confirmatory correlation analyses constrained to regions demonstrating main effects, for which correction was not applied. We also applied a whole-brain regression of α power on hypervigilance scores, followed by Monte Carlo multiple comparison correction. While the sensory hypothesis implicates a direct association between sensory disinhibition and hypervigilance symptoms, it is possible that this sensory pathology also contributes to other PTSD symptom clusters. However, correlations between reduced source-level α power and total PCL scores (S-RS/M-RS *r*s > −0.10/−0.14, *p*s > 0.41/0.25) or other subscale scores (*r*s > −0.16; *p*s > 0.19) indicate rather weak effects. We thus focused on the hypervigilance symptoms below. Trend-level and non-corrected effects will be reported but not further discussed.

## Results

### PTSD-related α power deficits in the DMN and VC

Validating intracranial source estimation of α oscillations, we observed “α blocking” by visual stimulation. Specifically, the contrast between S-RS versus M-RS (collapsed across groups) isolated α power reduction at M-RS across a large cluster (624 voxels) spanning bilateral visual cortices (peak voxel: −20, −100, 0; *t* = −4.50, *d *=* *1.09).

**Figure 1.**
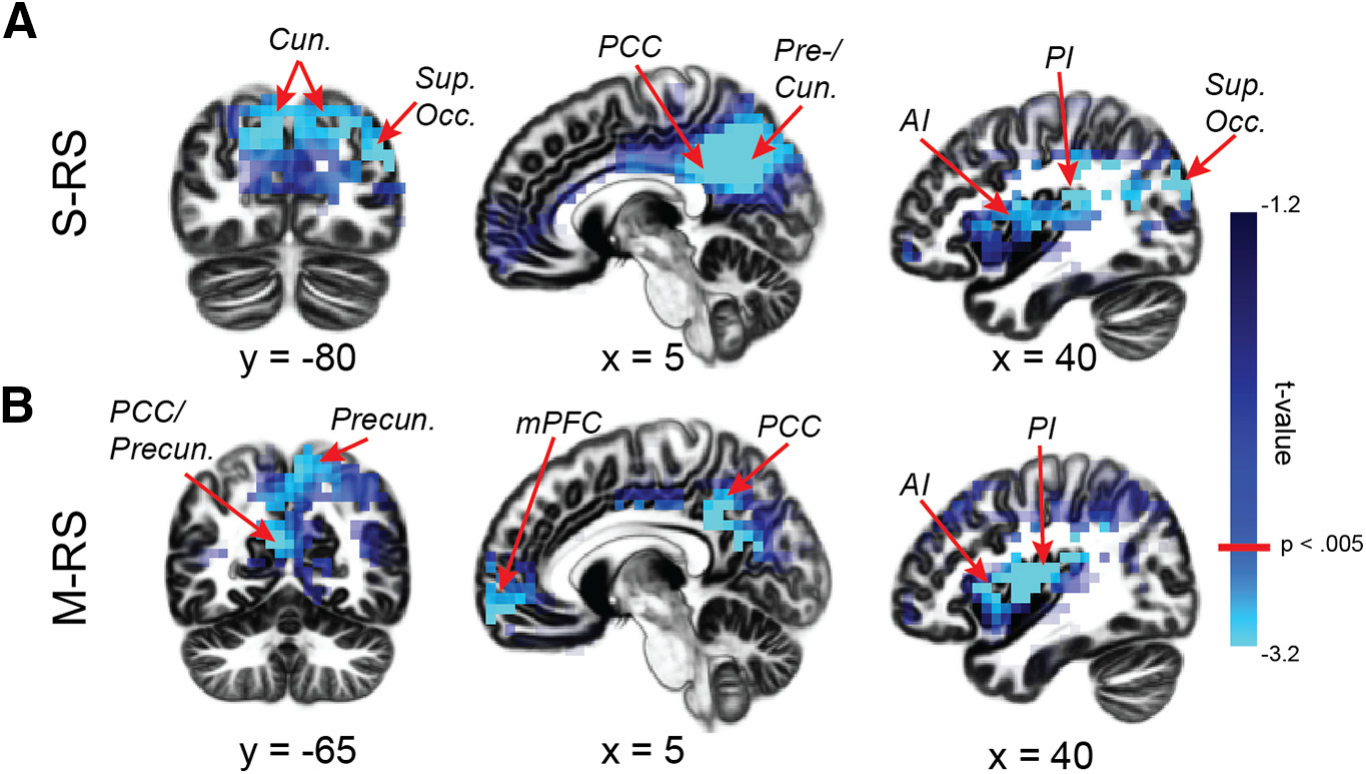
Group differences in α power. ***A***, During the S-RS, the PTSD group demonstrated reduced α power in the VC (the cuneus, precuneus, and superior occipital gyrus), the posterior DMN hub (PCC), and anterior and posterior insula. ***B***, During the M-RS, the PTSD group showed reduced α power in the VC (the cuneus and precuneus), both the anterior and posterior DMN hubs (mPFC and PCC), and anterior and posterior insula. Cun = cuneus; Precun = precuneus; AI = anterior insula; PI = posterior insula; Sup. Occ. = superior occipital gyrus.

Next, simple contrasts of group (PTSD vs controls) revealed α power deficits in PTSD across the visual and DMN ROIs at each state. The VC exhibited the strongest group effects: at S-RS, a large cluster (283 voxels) spanning bilateral cuneus (peaks: 5, −70, 30/−5, −70, 30; *t*s < −3.41, *d*s > 0.83), bilateral precuneus (peaks: 5, −60, 35/−5, −65, 25; *t*s < −3.75, *d*s > 0.92), and the right superior occipital gyrus (peak: 40, −80, 25; *t* = −3.90, *d*s > 0.96; Fig. 1*A*); at M-RS, a cluster (68 voxels) in the bilateral precuneus, overlapping the S-RS cluster (peaks: 5, −55, 50/−5, −55, 40; *t*s < −3.11, *d*s > 0.76; Fig. 1*B*). Regarding the DMN, α deficits emerged in PTSD in the bilateral PCC for both states (S-RS: 60 voxels; peaks: 15, −45, 40/−5, −50, 35; *t*s < −3.32, *d*s > 0.81; M-RS: 199 voxels; peaks: 5, −30, 25/−5, −30, 25; *t*s < −3.76, *d*s > 0.92; Fig. 1*A*,*B*), and the bilateral mPFC at M-RS only (88 voxels; peaks: 15, 60, −10/−15, 65, −15; *t*s < −3.10, *d*s 0.76; Fig. 1*B*). Additional α power deficits (whole-brain corrected) appeared in the bilateral insula [S-RS: right/left: 49/76 voxels; peaks: 35, −5, 20/−35, 20, 5 (Fig. 1*A*); M-RS: right/left: 136/25 voxels; peaks: 40, −5, 15/−30, 25, 5 (Fig. 1*B*); *t*s < −3.52, *d*s = 0.86]. No significant clusters emerged for enhanced α power in the PTSD group, even at a lenient threshold of *p* < 0.05. Finally, double contrasts of state (M-RS minus S-RS) and group (PTSD minus control) yielded no difference between groups (voxel-level *p*s > 0.11). These group effects are summarized in Table 2.

**Table 2:** Summary of group effects

Effects (PTSD < control)	State	Visual cortex	DMN
α Power	*S-RS*	Cun., Precun., Sup. Occ.	PCC
	*M-RS*	Precun.	PCC, mPFC
	*M-RS* - *S-RS*	*n. s.*	*n. s.*
α GC	*S-RS*	*n. s.*	*PCC→VC*
	*M-RS*	VC→PCC, *VC→mPFC*	PCC→mPFC
	*M-RS* - *S-RS*	VC→PCC, VC→mPFC	*n. s.*

Cun = cuneus; Precun = precuneus; Sup. Occ. = superior occipital gyrus; VC = visual cortex. Italicized ones were significant (*p *<* *0.05) before multiple comparison correction; all other effects survived correction.

Individual group contrasts of PTSD versus GAD or HC separately were then performed to substantiate the PTSD versus controls difference. As summarized in Table 3, concerning α power, these contrasts revealed essentially identical results as reported above. Moreover, the effect sizes were comparable (*d*s = 0.74–1.04) and survived correction for multiple comparisons. In addition, HC and GAD did not differ on any of these contrasts (*p*s > 0.39).

**Table 3:** Summary of individual group contrasts

	Contrast	S-RS	M-RS	M-RS – S-RS
α Power	PTSD vsHC	PCC/Precun. (20, –60, 35)(*t* = –3.00; *k* = 71) Cun./SOC (45, –80, 25) (*t* = –3.42;*k* = 71) R. Insula (35, –5, 15) (*t* = –3.18;*k* = 10)	PCC/Precun. (5, –55, 50) (*t* = –2.41; *k* = 121) mPFC (10, 65, 0) (*t* = –2.87; *k* = 10) R. Insula (45, –5, 15) (*t* = –2.98; *k* = 10)	n.s.
	PTSD vsGAD	PCC/Precun. (15, –50, 40)(*t* = –3.31; *k* = 112) Cun. (5, –65, 15) (*t* = –2.88;*k* = 112) L. Insula (–30, 25, 0) (*t* = –4.29;*k* = 30)	PCC/Precun. (15, –45, 40) (*t* = –3.31; *k* = 102) mPFC (–15, 65, –15) (*t* = –3.31; *k* = 95) R./L. Insula (60, –15, 30/–30, 25, 5)(*t* = –3.96/–3.58; *k* = 64/65)	n.s.
α Connectivity	PTSD vsHC	PCC→VC^*^ (*t* = –1.72, *p* = 0.046)	PCC→mPFC (*t* = –2.68, *p* = 0.010) VC→PCC (*t* = –2.36, *p* = 0.023)	VC→PCC^*^ (*t* = –1.87,*p* = 0.034)
	PTSD vsGAD	PCC→VC^*^ (*t* = –1.83, *p* = 0.037)	PCC→mPFC (*t* = –2.68, *p* = 0.010) VC→PCC (*t* = –3.30, *p* = 0.002) mPFC→PCC (*t* = –3.25, *p* = 0.003)	VC→PCC^*^ (*t* = –1.87,*p* = 0.035) VC→mPFC (*t* = –2.29,*p* = 0.027)

All effects survived FDR *p *<* *0.05.

* one-tailed. Peak MNI coordinates (*x*, *y*, *z*) are provided, along with cluster sizes (k). SOC = superior occipital gyrus; R/L = right/left.

### PTSD-related α connectivity deficits within and between the DMN and VC

Like the power analyses above, we first assessed the effect of State (S-RS vs M-RS) on α-frequency connectivity (collapsed across the groups). We observed reduced bidirectional α connectivity from the S-RS to the M-RS within the DMN: PCC→mPFC (M-RS minus S-RS; *t* = −3.49, *p *=* *0.001, FDR *p *<* *0.05, *d *=* *0.85) and mPFC→PCC (*t* = −3.14, *p *=* *0.003, FDR *p *<* *0.05, *d *=* *0.76), suggesting disrupted DMN connectivity in general by salient visual input (at M-RS).

**Figure 2.**
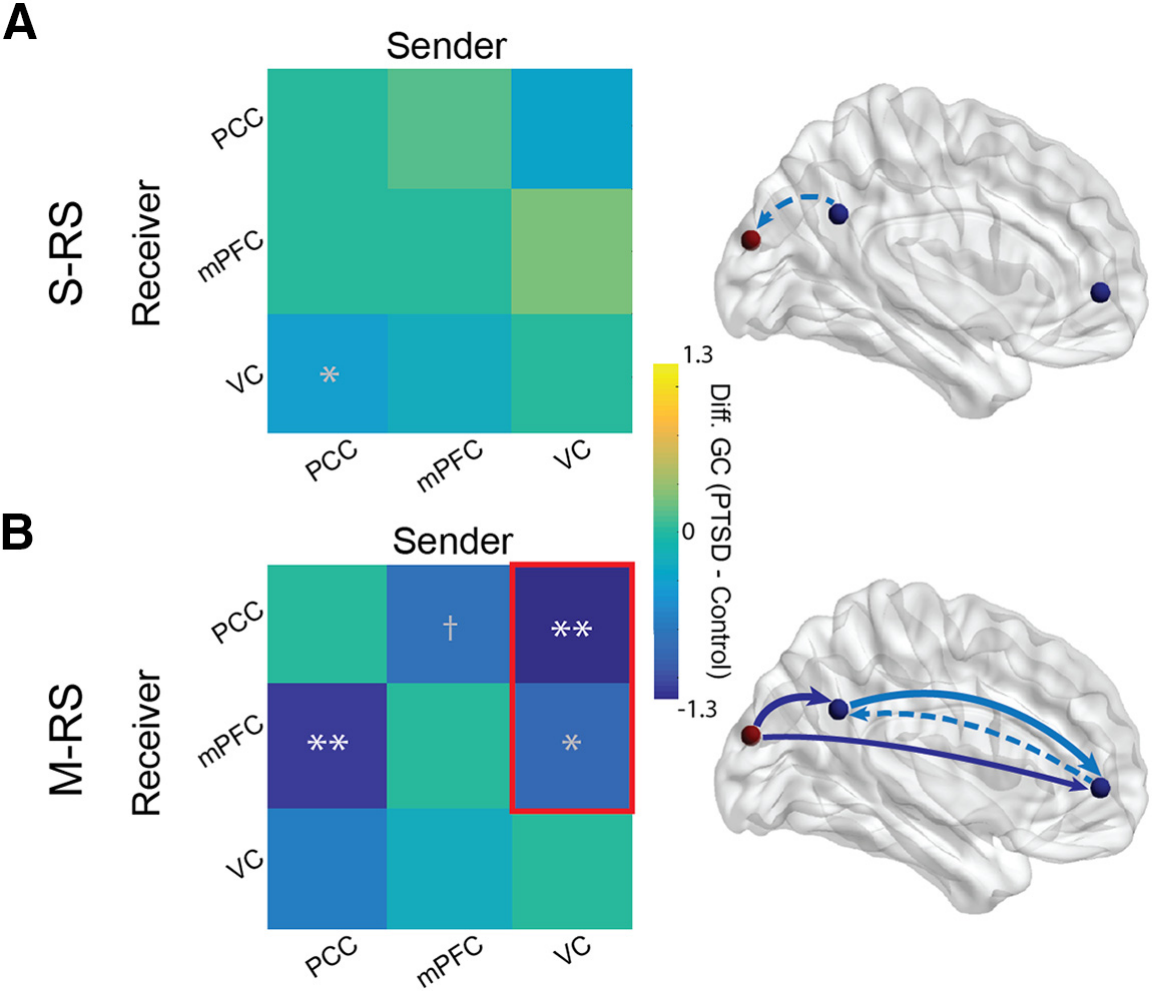
Group differences in α connectivity. Left column, Matrices of group differences PTSD minus controls in directed α-frequency connectivity (GC) showed (***A***) reduced PCC→VC α connectivity (albeit not FDR corrected) during the S-RS; and (***B***) reduced PCC→mPFC α connectivity during the M-RS and, as enclosed in a red box, more reduction from the S-RS to the M-RS in VC→PCC and VC→mPFC α connectivity. Right column, Schematic presentations of group differences in connectivity during the S-RS (***A***) and M-RS (***B***), with solid and dotted arrows reflecting connections surviving and not surviving FDR correction, respectively. Arrows in light blue and dark blue reflect significant effects from simple group contrasts and double contrasts of state and group, respectively. Our discussion focused on the effects surviving the multiple comparison correction; *p <* *0.05, **p <* *0.01, †*p *<* *0.1; white * = FDR corrected; gray * = not FDR corrected. VC = visual cortex.

**Figure 3.**
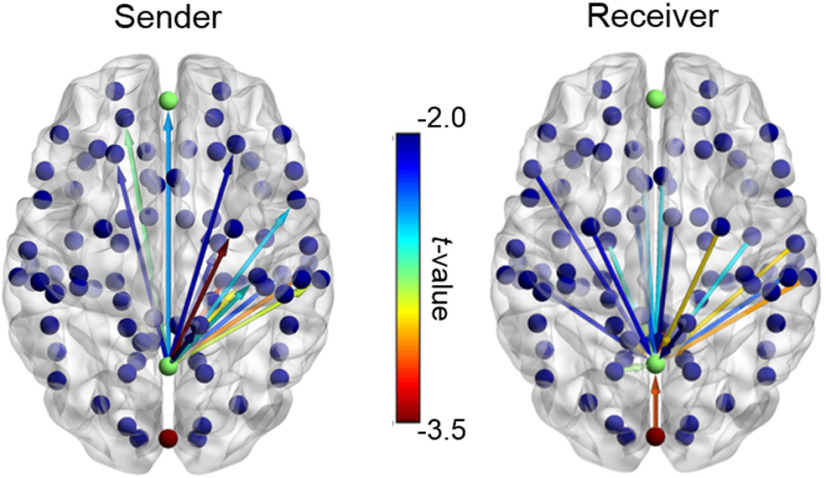
Whole-brain PCC connectivity maps during the M-RS. Connectivity for all Brodmann areas (BAs) with the PCC as the sender and receiver (*p* < 0.05). Only the PCC→BA 22 connectivity (dark red) survived whole-brain correction (FWE *p* = 0.0006).

Next, simple contrasts of Group for the S-RS revealed reduced α connectivity from the PCC to the VC (PCC→VC) in PTSD (vs controls; *t* = −2.26, *p *=* *0.027, *d *=* *0.55; Fig. 2*A*), albeit failing FDR correction. No other effects emerged during this state (*p*s > 0.34). For the M-RS, the PTSD group (vs controls) demonstrated reduced connectivity within the DMN, including PCC→mPFC (*t* = −2.82, *p *=* *0.008, FDR *p *<* *0.05, *d *=* *0.69; Fig. 2*B*) and, at a trend level, mPFC→PCC (*t* = −1.91, *p *=* *0.064, *d *=* *0.47). The PTSD group demonstrated additional deficits in α connectivity from the VC to both DMN ROIs at M-RS: VC→PCC (*t* = −3.06, *p *=* *0.004, FDR *p *<* *0.05, *d *=* *0.75) and VC→mPFC (*t* = −2.05, *p *=* *0.049, *d *=* *0.50; albeit not FDR corrected). No effect appeared in the opposite (DMN→VC) direction (*p*s > 0.12). We further explored whole-brain connectivity with the PCC seed during the M-RS between the PTSD and control groups (Fig. 3). At the familywise threshold, correcting for the 84 Brodmann areas examined (FWE *p *=* *0.0006), only one additional area emerged with attenuated connectivity from the PCC to the right associative auditory cortex (superior temporal gyrus, Brodmann’s area 22; *t* = −3.93, *p *=* *0.0003) in the PTSD group at MRS. This effect thus aligned with the VC-PCC dysconnectivity in PTSD above.

Double contrasts of State (M-RS minus S-RS) and Group (PTSD vs control) revealed different effects of state (i.e., visual stimulation) on connectivity between the groups. Greater connectivity reduction from S-RS to M-RS appeared in the PTSD (vs control) group in VC→PCC (*t* = −2.22, *p *=* *0.032, *d *=* *0.54) and VC→mPFC (*t* = −2.18, *p *=* *0.036, *d *=* *0.53). Specifically, from S-RS to M-RS, significant connectivity reduction emerged in VC→PCC (*t* = −2.84, *p *=* *0.011, FDR *p *<* *0.05, *d *=* *0.69) and VC→mPFC (*t* = −3.04, *p *=* *0.007, FDR *p *<* *0.05, *d *=* *0.74) in the PTSD group, but none in the control group (*p*s > 0.628). These group effects are also summarized in Table 2.

Finally, individual group contrasts were performed to substantiate the PTSD versus controls difference. Again, these specific contrasts revealed consistent results to those from the main analyses (Table 3). A notable difference was that, owing to the reduced group size, the PCC→VC deficit in PTSD at S-RS survived one-tailed tests only (PTSD vs HC: *t* = −1.72, *p *=* *0.046 one-tailed, *d *=* *0.52; vs GAD: *t* = −1.83, *p *=* *0.037 one-tailed, *d *=* *0.53), as did the VC→PCC deficit in the double contrast (PTSD vs HC, *t* = −1.87, *p *=* *0.034 one-tailed, *d *=* *0.57; PTSD vs GAD, *t* = −1.87, *p *=* *0.035 one-tailed, *d *=* *0.55). However, given the strong a priori hypothesis of deficient α connectivity in PTSD, these tests could provide support to the hypothesis. The double contrast on VC→mPFC α connectivity showed a significant deficit in PTSD in comparison to GAD (*t* = −2.29, *p *=* *0.027, *d *=* *0.67) but only a marginal deficit in comparison to HC (*t* = −1.50, *p *=* *0.071 one-tailed, *d *=* *0.46).

These group-specific contrasts also revealed additional results: at M-RS, the GAD (vs PTSD or HC) group demonstrated increased α connectivity from mPFC→PCC (GAD vs PTSD: *t *=* *3.25, *p *=* *0.003, *d *=* *0.95; GAD vs HC: *t *=* *2.65, *p *=* *0.012, *d *=* *0.82), suggesting GAD-specific augmentation in this α connectivity. No other differences emerged between the HC and GAD groups (*p*s > 0.411).

### Clinical associations

**Figure 4.**
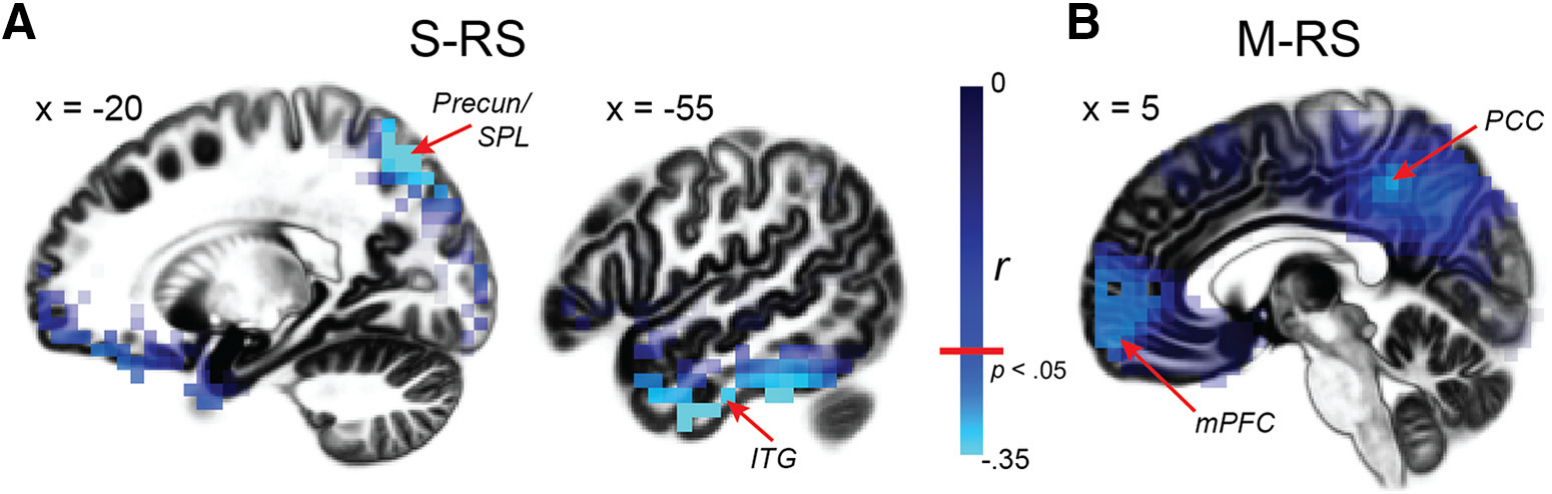
Clinical associations between α power and hypervigilance. Whole-brain correlation maps of α power and hypervigilance indicated negative correlations in both the dorsal (i.e., SPL, precuneus) and ventral (i.e., ITG) visual cortices during the S-RS (***A***) and both DMN hubs (mPFC and PCC) during the M-RS (***B***). SPL = superior parietal lobule; ITG = inferior temporal gyrus.

We then performed correlation analyses, regressing power or connectivity values on symptom severity in hypervigilance. Confirmatory analyses (constrained to the regions identified in the main contrasts) revealed negative associations between hypervigilance and α power in the precuneus/superior parietal lobule at S-RS (39 voxels; peak: −20, −75, −55; *r* = −0.49, *p *<* *0.005; Fig. 4*A*) and the DMN at M-RS: PCC (13 voxels; peak: 5, −45, 45; *r* = −0.28, *p *<* *0.05) and mPFC (50 voxels; peak: −10, 65, −15; *r* = −0.30*, p *<* *0.05; Fig. 4*B*). Whole-brain regression analysis further revealed negative associations with α power in the ventral VC at S-RS (left inferior temporal gyrus: 13 voxels; peak: −55, −5, −40; *r* = −0.40, *p *<* *0.005 whole-brain corrected; Fig. 4*A*). There was no significant correlation between hypervigilance and α connectivity (*p*s > 0.20).

## Discussion

Source-level analysis of RS α oscillations isolated α (power and connectivity) deficits in the DMN and VC in PTSD, especially during strong visual stimulation. In support of our first hypothesis, α deficits in the DMN, the PTSD group demonstrated reduced α power in the posterior DMN hub (the PCC) at both S-RS and M-RS as well as the anterior hub (the mPFC) at M-RS, accompanied by reduced DMN (PCC→mPFC) α-frequency connectivity at M-RS. In support of the second hypothesis, α deficits in the VC, the PTSD group exhibited reduced α power in the VC at both states. Importantly, joining α deficits in the two neural systems, diminished α-frequency connectivity from the VC to the DMN was observed in the PTSD group at M-RS. Finally, α power deficits in the DMN and VC directly correlated with symptom severity of hypervigilance. Therefore, linking anomalies in DMN and α activity in PTSD, the current results indicate interrelated α deficits in the DMN and VC, implicating visual-cortex-DMN α dysrhythmia in the neuropathology of PTSD.

Neural oscillations actively participate in mental activities by modulating local neuronal excitability and mediating long-range neural communication (Buzsáki and Draguhn, 2004; Buzsáki et al., 2013). Aberrant resting-state (intrinsic) neural oscillations (e.g., thalamocortical dysrhythmia) in neuropsychiatric disorders has been increasingly recognized (Llinás et al., 1999; Schulman et al., 2011; Vanneste et al., 2018), promoting the transdiagnostic conceptualization of “oscillopathies” for these disorders (Basar, 2013; Buzsáki et al., 2013). Advancements in neural computational algorithms (such as eLORETA) have permitted intracranial source estimation of neural oscillations in hdEEG recordings, providing important insights into oscillatory dysrhythmia in multiple disorders, e.g., schizophrenia (Canuet et al., 2011; Di Lorenzo et al., 2015), depression (Whitton et al., 2018), and PTSD (Imperatori et al., 2014). As validation of our hdEEG source analysis, we confirmed a strong “α blocking” effect of visual stimulation by demonstrating extensive α power reduction in bilateral visual cortices from the S-RS (minimal visual input) to the M-RS (strong visual input). By contrast, no other regions emerged from this contrast, highlighting the sensitivity and specificity of this source analysis of α oscillations.

Our source-level group analysis further identified α deficits within and between the VC and the DMN in PTSD. Concerning the VC, α oscillations are known to mediate visual cortical inhibition, and accordingly, α power correlates inversely with visual cortical activity (Klimesch et al., 2007; Palva and Palva, 2007; Jensen and Mazaheri, 2010; Lange et al., 2013). In the PTSD group, reduced α power in the VC was extensive and enduring across states. Without visual stimulation (at S-RS, reflecting intrinsic activity), α deficits spanned a large cluster over the cuneus, precuneus, and superior occipital gyrus, suggesting wide-spread, intrinsic neural disinhibition and hyperactivity across primary and secondary visual cortices in PTSD. This sensory cortical disinhibition aligns with extant electrophysiological evidence of impaired sensory gating and sensory cortical hyperactivity (to simple, neutral stimuli; Morgan and Grillon, 1999; Neylan et al., 1999; Stewart and White, 2008; Javanbakht et al., 2011) and behavioral disturbances in sensory filtering/gating and response in these patients (Stewart and White, 2008; Engel-Yeger et al., 2013). With strong visual input at M-RS, α power reduction was particularly localized to the parietal VC (primarily bilateral precuneus). Given that this region is strongly involved in visual spatial attention and perception (Corbetta et al., 1995; Behrmann et al., 2004; Corbetta and Shulman, 2011) and that the M-RS condition mimics a real-life environment with salient sensory information, this α deficit (reflective of disinhibited visual spatial attention) can underlie hypervigilance in PTSD, expressed as excessive alertness to and scanning of the environment (Conoscenti et al., 2009).

As mentioned above, α oscillations also play a role in long-range neural communication and resting-state α power correlates positively with DMN activity (Mantini et al., 2007; Jann et al., 2009; Sadaghiani et al., 2010; Knyazev et al., 2011; Mo et al., 2013; Samogin et al., 2019). The α deficits in DMN hubs thus dovetails the extant literature citing both deficient α activity (Clancy et al., 2017; Clancy et al., 2020) and DMN hypoactivity in PTSD (Koch et al., 2016; Akiki et al., 2018). Notably, resting-state β and θ oscillations have also been found to be associated with DMN activity (Laufs et al., 2003; Mantini et al., 2007; Scheeringa et al., 2012). However, prior sensor-level analyses have not revealed PTSD-related anomalies in the β or θ frequencies (*p*s > 0.16; Clancy et al., 2017). Future research is warranted to examine the other oscillatory activities in various states or tasks to elucidate their contribution to PTSD pathology.

The fact that DMN α power reduction extended from the PCC only at S-RS to both the PCC and mPFC at M-RS accentuates the particular DMN vulnerability in PTSD in a sensory-rich environment. In addition, as α oscillations synchronize activity and facilitate coherence across regions, reduced α power in these key DMN hubs could further suggest compromised communication across the network in PTSD. Indeed, connectivity analyses revealed reduced PCC→mPFC α connectivity at M-RS in the PTSD group. This hypoconnectivity between the DMN hubs highlights impaired communication within the core architecture of the DMN. Clinically, DMN α deficits in both local power and interhub connectivity, especially acute in a sensory-rich environment, could contribute to difficulty in maintaining “tranquility” or “rest” (Buckner et al., 2008; Abdallah et al., 2017; Akiki et al., 2018) and avoidance of sensory stimulation in patients with PTSD (Stewart and White, 2008; Engel-Yeger et al., 2013).

Our manipulation of visual stimulation between the two states revealed a direct link between α deficits in the VC and the DMN. Consistent with general DMN susceptibility to salient sensory input, we confirmed a general reduction (in the entire sample) in bidirectional α connectivity between the DMN hubs (PCC→mPFC and mPFC→PC) as visual input increased from the S-RS to the M-RS. Beyond that, in the PTSD (but not control) group, increased visual stimulation (from S-RS to M-RS) further diminished visual cortical α connectivity to both DMN hubs (VC→PCC and VC→mPFC). As mentioned above, α oscillations in the sensory cortex mediate sensory inhibition, such that this VC→DMN α projection would serve to protect the DMN by blocking sensory afferents to the network. This notion is supported by prior work demonstrating a modulatory role of α oscillations in the connectivity within the sensory (visual) cortex and between the VC and DMN hubs (Scheeringa et al., 2012). While this protective inhibitory process withstood the increased visual input at M-RS in the control group, it broke down among patients with PTSD, suggesting impaired gating of sensory entry to (i.e., compromised protection of) the DMN. Exploratory correlation analyses further indicated a close correlation between VC→PCC and PCC→mPFC α connectivity at M-RS (*r *=* *0.31, *p *=* *0.013), highlighting a mechanistic link between these two pathways. That is, disinhibited visual cortical propagation to the PCC, in the presence of strong environmental input, could further impair PCC-driven α synchronization with the mPFC, worsening DMN dysfunction in PTSD. Finally, to rule out the alternative explanation that emotional content (beyond visual stimulation) could contribute to the M-RS effects, we compared RS EEG data acquired before and after 5-min presentation of negative international affective picture system (IAPS) pictures from an independent sample of healthy individuals (*N *=* *45). There was no change in α GC (*t *=* *0.032, *p *=* *0.974), suggesting that in the absence of visual stimulation, simple affective effects would not result in α connectivity change. Nonetheless, we cannot fully exclude the combined effects of emotion and visual stimulation. That is, the emotional content of visual stimuli may influence α GC differently among the three groups. Future studies are warranted to isolate the simple effects of visual stimulation by including neutral images alone.

Together, current findings implicate a core visual-cortex-DMN system of α dysrhythmia in PTSD. The critical role of visual cortical α deficits in this pathology lends credence to a sensory hypothesis of PTSD centered on sensory cortical disinhibition (Clancy et al., 2017, 2020; Li, 2019) and bottom-up accounts of PTSD in general (Nicholson et al., 2017; Badura-Brack et al., 2018). Childhood trauma, a common PTSD risk factor, has been associated with various aberrations in the sensory cortex and sensory pathway (Teicher et al., 2016), adding to the support for this sensory hypothesis. Indeed, 36% of the current PTSD sample reported childhood trauma. That said, the means (and standard deviations) of α power and connectivity were highly comparable between the childhood trauma subgroup and the rest of the PTSD group, implicating this sensory pathology across trauma types.

This core visual-cortex-DMN system of α dysrhythmia was reinforced by the specificity of α deficit sources. Despite the use of whole-brain analyses, group differences in α power and clinical associations between α power and hypervigilance were localized to the VC and the DMN only (except for the associative auditory cortex and insula as discussed below). While DMN hypoactivity is known to be associated with PTSD symptom severity (Sripada et al., 2012; Miller et al., 2017a,b; Akiki et al., 2018), the clinical association in the VC highlights an additional pathway linking visual cortical disinhibition to PTSD symptoms. Notably, the associations with hypervigilance implicate not only both the dorsal (i.e., the parietal cortex) and ventral visual cortices (i.e., the inferior temporal gyrus) but also the higher-order regions with strong interactions with limbic and frontal regions. As the ventral VC underpins visual object perception, its involvement here aligns with the fact that hypervigilance in PTSD is associated with hyperactivity and hypersensitivity to (threat and neutral) sensory cues (Ehlers and Clark, 2000; Hayes et al., 2012), in addition to excessive spatial scanning engaging the dorsal VC. In light of the “sentinel hypothesis” implicating the DMN in sustained monitoring of the environment (i.e., sensory vigilance; Buckner et al., 2008; Andrews-Hanna, 2012), we surmise this visual-cortex-DMN α dysrhythmia in PTSD may fuse the impairment in “sentinel” function (of the DMN) and in sensory cortical inhibition (via α oscillations), resulting in a pathologic state hypervigilance.

While the insula was not an a priori ROI in the current study, expansive α deficits spanning the posterior and anterior insula emerged in PTSD following whole-brain correction. The insula is a highly heterogeneous structure, with the posterior portion receiving strong sensory afferents and the anterior portion densely connected with limbic and prefrontal regions as a key node of the salience network/SN (Craig, 2002; Critchley, 2004; Seeley et al., 2007; Menon and Uddin, 2010). Abnormal insular and SN activity has been repeatedly observed in patients with PTSD (Lanius et al., 2015; Koch et al., 2016), and α suppression via neurofeedback can increase SN activity (Ros et al., 2013). Our data provide preliminary evidence of insular α dysrhythmia in PTSD, potentially underlying some of the insular anomalies. Interestingly, our exploratory whole-brain connectivity analyses further isolated an additional region, the right superior temporal gyrus, showing attenuated connectivity with the DMN (i.e., PCC) in PTSD. This area represents the associative auditory cortex and a primary source of auditory α (“tau”) oscillations (Lehtelä et al., 1997; Billig et al., 2019). This finding thus further bolsters the pathology of sensory-cortex-DMN α dysconnectivity in PTSD, extending beyond our a priori focus on the VC to other sensory modalities.

We included patients with GAD as an additional control condition to rule out general effects of anxiety and arousal on α activity. Previous sensor-level analysis of α oscillations revealed no α deficits in GAD (Clancy et al., 2017), which directed us to combine patients with GAD and HCs into one control group in the source-level analysis. Nonetheless, we systematically explored group-specific effects. While the results essentially echoed the main results, we noticed interesting findings of enhanced DMN (mPFC→PCC) α connectivity in GAD at M-RS, in comparison to the PTSD and HC groups. We surmise that this mPFC→PCC hyper-connectivity could heighten DMN functioning in GAD, supporting the hallmark symptoms of self-referential rumination and worry in these patients.

Integrative neuroimaging and electrophysiological research have promoted the idea that α oscillations sustain and facilitate DMN functioning by synchronizing spontaneous activity across the network and suppressing sensory cortical propagation. Translating these mechanisms to the neuropathology of PTSD, we confirmed interconnected α deficits in the DMN and VC in patients with PTSD, which are further associated with symptoms of hypervigilance. Therefore, the current findings provide the first evidence of visual-cortex-DMN α dysrhythmia in PTSD, presenting a unifying neural underpinning of sensory disinhibition, DMN dysfunction, and hypervigilance in this disorder. The specification of this α dysrhythmia further isolates a novel therapeutic target, promoting network-based interventions (Lanius et al., 2015) using brain stimulation of α oscillations (Clancy et al., 2018) in the visual-cortex-DMN system as a new line of treatment for PTSD.

## Synthesis

Reviewing Editor: Bradley Postle, University of Wisconsin Decisions are customarily a result of the Reviewing Editor and the peer reviewers coming together and discussing their recommendations until a consensus is reached. When revisions are invited, a fact-based synthesis statement explaining their decision and outlining what is needed to prepare a revision will be listed below. The following reviewer(s) agreed to reveal their identity: René Scheeringa.

Here is the full text of the two reviews:

Reviewer 1

This is an interesting and important paper, comparing alpha power and connectivity in PTSD and controls in both resting and visual stimulation conditions. Findings included lower alpha power in PTSD in visual cortices, DMN regions, and insula, mostly independent of state. These reductions correlated with hypervigilance. There were no state by group interactions for alpha power. When presented with visual stimuli, the PTSD group had lower connectivity than the HC group within the DMN, and from visual cortex to the DMN. Additionally, there was a state by group interaction; the PTSD group showed a stronger reduction in connectivity from visual cortex to DMN when switching from no visual input to visual input. The paper contributes to a growing body of literature demonstrating sensory processing abnormalities in PTSD.

The introduction is clearly written, easy to follow, comprehensively cited, and a pleasure to read. I do have a few concerns regarding the analysis.

My first major concern has to do with the combination of the HC and GAD groups. The introduction provides a rationale for including the GAD group (“The GAD group was included to rule out effects of general anxiety and hyperarousal that would confound resting-state neural oscillations”), but then the analysis unexpectedly collapses the HC and GAD groups. This is confusing to read and the justification is unclear until very late in the paper. I think the introduction should provide some rationale for contrasting the PTSD group against a group of mixed HC and GAD, or the analysis should be an analysis of all 3 groups separately, but not both. The explanation about combining the HC and GAD groups is provided very late in the methods section, and this is also confusing. I think the paper would be much stronger with a three-group design. If there are no differences in alpha activity between GAD and HC, this strengthens the argument that effects are not merely due to anxiety and it is worth developing this in this manuscript. This analysis is presented in the supplement, but without effect sizes or comments on significance corrected for multiple comparison. Reporting the t’s is not sufficient, I’d like to know that effect sizes are comparable to those reported in the main text, and whether these survive correction for multiple comparisons.

My second major concern involves the visual stimulus. What was the rationale for including positive and negative IAPS pictures, as well as neutral pictures? If the key difference between the S-RS and M-RS states is supposed to be strong visual stimulation, it’s not clear to me why strong affective stimulation was also delivered. I’m concerned that activation of emotion-sensitive regions in response to affective pictures might drive these results. Do the results hold up if only neutral pictures are analyzed?

Additional concerns:

1. How many of the controls were trauma-exposed? How were the healthy controls recruited? Were participants compensated?

2. A comprehensive description of inclusion/exclusion criteria for each group is needed. (TBI? etc.)

3. Please provide trauma types. I am also curious about childhood maltreatment in this sample, esp given work by Marty Teicher and others showing changes in visual pathways in people with childhood trauma exposure (a common risk factor for PTSD).

4. Why were diagnoses made using SCID-5, but the PCL for DSM-IV was used? The criteria for PTSD differ substantively from DSM-IV to 5.

5. Regarding the supplemental analyses, it’s unclear to me why the t values are negative for the comparison of PTSD vs HC and positive for PTSD vs GAD. Shouldn’t these be in the same direction? The text lists the p for the GAD clusters as e.g. t’s < 3.29, which would mean small (non-significant) t’s. I think maybe there are some typos here.

6. The correlations with hypervigilance are interesting but are interpreted as if specificity has been shown, which it hasn’t. Are correlations with total PCL significant? How about other symptom clusters? If the argument is that these results are specifically related to hypervigilance, the authors would need to know that the findings are not related to non-hypervigilance-related symptoms.

7. Typo on line 385 (doral -> dorsal)

8. Finally please note that I feel that this review was not completely blinded, because I think the authors’ references to their prior paper demonstrating no differences between the HC and GAD samples revealed their identity as I was previously familiar with that paper. I don’t know anyone personally and perceive no conflict of interest. I checked with the editors re: the unblinding, and they suggested that I proceed. Nevertheless, just want to make everyone aware that this review was not what I would consider completely blinded.

Reviewer 2

Since I have no expertise on PTSD or anxiety disorders I will refrain from commenting on these aspects of the article.

General remarks

In general I think the methodology used in this manuscript is appropriate and well conducted. My main concerns are with the analyses that are not carried out and that I think would benefit the interpretability of the findings. These concerns predominantly pertain to the frequency and anatomical specificity of findings. Also, the theoretical underpinnings for conducting the analyses seem to me to be weaker than the authors claim.

Specific comments

1) The author(s) make the case that the base their approach in (in part) on the observed link between alpha power and the default mode network (DMN). The alpha oscillations however are not the only frequency bands that have been related to DMN activity and connectivity. There is substantial evidence that (frontal) theta (Scheeringa et al., 2008: International journal of psychophysiology; 2009: Neuroimage) and beta oscillations (Laufs et al., PNAS 2003)) are as well related to the DMN. Much of the interpretation of the results are predicated on the assumption that the alpha-DMN link is of particular interest for PTSD. This would not be the case if similarly strong effects would be observed for theta and beta power, both for the differences in source localized power as well as directional connectivity based on granger causality.

2) For the power analysis the depicted Figure 1, the regions observed are indeed in line with the nodes of the DMN in the medial wall of the cortex. For the directed connectivity analysis, only two of the MDN nodes and one node in Visual cortex are used. It therefore lacks the same anatomical specificity as in Figure 1. Given the inherent low resolution of EEG sources analysis, it cannot be excluded that the effects are not related to visual cortex and DMN. For the significant connections observed in Fig2., I would therefore suggest to do a brain wide analysis using the same seed (sender). This should give an indication whether the observed effects are indeed DMN network specific.

3) Furthermore, triggered by an inaccurate citation of my own work on line (61-62), I further checked the findings of the articles cited in relation to the same statement.

Citation (lines 61-62:

"Importantly, converging evidence has linked alpha oscillations to DMN activity, advancing the notion that the DMN could be organized and maintained by long-range synchronization of alpha oscillations(Laufs et al., 2003; Mantini et al., 63 2007; Jann et al., 2009; Knyazev et al., 2011; Scheeringa et al., 2012; Mo et al., 2013).”

Many of these studies are EEG-fMRI, and most of these studies looking at correlations between neural oscillations and BOLD. They do show correlations between BOLD activity and neural oscillations, but this does not necesarily leed to the idea that alpha underlies that the DMN could be organized and maintained by long-range synchronization of alpha oscillations. In Scheeringa et al. (2012) , the DMN does correlate with positively posterior alpha (as is shown in Mo et al as well) , but fMRI based (negative and positive) connectivity in general becomes weaker with when alpha power is higher and evidence for a change in connectivity between visual and DMN connectivity is absent or weak, and if present also goes in the opposite direction. The cited study by Laufs actually shows the strongest relation between beta and the DMN. The study by Jann et al., doe seem to be more in line with the statement, but overall the cited support for the notion that long-range alpha band synchronization underlies the DMN is not as strong as the authors suggest here. This does not in itself invalidate the results obtained here, but to me it seems to be something that needs to be addressed. It does imply the authors should make a stronger effort showing that the observed results are more frequency and anatomically specific, as I have pointed out above.
